# An Unambiguous Delay-And-Multiply Acquisition Scheme for GPS L1C Signals

**DOI:** 10.3390/s18061739

**Published:** 2018-05-28

**Authors:** Duk-Sun Shim, Jin-Seok Jeon

**Affiliations:** School of Electrical and Electronics Engineering, Chung-Ang University, 84 Huksuk-ro, Dongjak-ku, Seoul 06974, Korea; cjs2506@cau.ac.kr

**Keywords:** GPS, L1C signal acquisition, delay-and-multiply approach, ambiguity problem

## Abstract

The GPS provides positioning information almost anytime and anywhere on Earth, regardless of the weather conditions, and has become an essential technology for positioning and navigation. As a modernization program, the fourth civil GPS signal, denoted as L1C, will be transmitted from Block III satellites. One distinction of the L1C signal from the former signals is the use of binary offset carrier (BOC) modulation, which is necessary for compatibility and the reduction of interference between the legacy L1 signal and L1C signal, despite their use of the same carrier frequency. One drawback of using BOC modulation is the ambiguity problem, which comes from the multiple peaks in the correlation function and causes difficulties finding the code phase in the acquisition process. In this paper we suggest two delay-and-multiply (DM) methods for the L1C signal to solve the ambiguity problem. For the DM acquisition schemes we suggest the optimal delay time for the delay signal, and prove that the correlation function of the received DM signal and the generated DM signal has a triangular shape, as seen in the legacy GPS L1 signal. The noise characteristics of the decision variable are obtained and the performance of the DM acquisition scheme is given in terms of the probability of detection, and compared with that of the conventional method. We provide the procedure to find the Doppler frequency after obtaining the code phase through the proposed DM method.

## 1. Introduction

The Global Positioning System (GPS) has become an essential technology for positioning, navigation, and timing after the full operational capability (FOC) in early 1995, and GPS modernization has now been in progress since January 1999 [1,2,3]. As a modernization program, two civil signals have been added at the L2 and L5 frequencies, which are denoted as the L2C and L5 signals, respectively [1]. The L2C signal has been transmitted from the GPS Block IIR-M satellite since September 2005, while the L5 signal from GPS Block IIF satellite has been transmitted since May 2010 [4,5]. The fourth civil signal, denoted as L1 Civil (L1C) signal, will be transmitted from the next generation satellites (GPS Block III) on the L1 carrier frequency [6,7]. One feature of the L1C signal is the use of binary offset carrier (BOC) modulation, which enables compatibility, and reduces interference between the legacy L1 signal and L1C signal, despite their use of same carrier frequency. 

The BOC modulation is also used in Galileo [8,9], the European Global Navigation Satellite System (GNSS) that is like the GPS of the United States, and enables Galileo E1 open service (OS) signal to use the same GPS L1 carrier frequency. The BOC modulation results in split spectrum signal, and thus enables frequency sharing [10,11,12,13]. After many years of research, MBOC (multiplexed BOC) is applied to GNSS, such that composite BOC (CBOC) is used for Galileo E1 OS signal, and time multiplexed BOC (TMBOC) for GPS L1C signal. GPS L1C signal consists of data and pilot channels with power ratio (75%/25% for the pilot/data) [14], where the data channel uses *BOC*_(1,1)_ modulation, and the pilot channel uses TMBOC modulation.

One drawback of using BOC modulation are the multiple peaks which appear in the correlation between the received signal and the generated signal, complicating the signal acquisition and tracking process, and termed the ambiguity problem. To deal with the BOC modulation, many techniques have been suggested [9,14,15,16,17], and several unambiguous methods, such as the “BPSK-like” technique, sub-carrier phase cancellation (SCPC) technique, and bump-jumping technique, have been suggested to deal with the ambiguities of the traditional BOC modulation [9,14]. Recently, the delay-and-multiply (DM) concept has been suggested for unambiguous acquisition of Galileo E1 OS signals in [18,19,20].

The DM concept exists from early GNSS history [3], but has not been studied in depth, since the DM method causes the multiplication of noise, which greatly deteriorates the signal acquisition performance. However, one advantage of the DM concept is the elimination of the Doppler frequency. That is, the DM method can reduce the two-dimensional acquisition process to a one-dimensional search. The Doppler frequency can be obtained after finding the code phase first. The DM concept also eliminates the effect of data symbol transition [19], which comes from the same length of the data symbol and the period of ranging code, so the non-coherent integration can be greatly increased.

The acquisition problem for GPS L1C signal has been studied in [21,22,23,24], but it is hard to find any research on the ambiguity problem for the GPS L1C signal. The DM acquisition method is attractive for the GPS L1C signal despite the multiplication of noise, since the L1C signal has a severe ambiguity problem and long search time, because of its long 10 ms ranging code. Even though [18,19,20] used the DM method in the Galileo E1 OS signal, which uses CBOC modulation, the GPS L1C signal uses TMBOC modulation. Thus the DM approach for the GPS L1C signal is a new problem and it is necessary to find optimal delay and the generated DM scheme again for the GPS L1C signal.

In this paper, we propose new DM acquisition schemes for the GPS L1C signal to eliminate the ambiguity of the correlation function between the received signal and the generated signal. We prove that the correlation function of our proposed scheme has a triangular shape, which completely removes the ambiguity problem, and analyze the performance of the proposed DM method using the signal-to-noise ratio (SNR) of the decision variable and the probability of detection. We also add the procedure to find the Doppler frequency for tracking the L1C signal.

The rest of the paper is organized as follows: Section 2 defines the GPS L1C signal and its DM signal, while Section 3 provides the result of this paper, such as new DM acquisition schemes for GPS L1C signal and the noise characteristics of the proposed DM method with performance analysis. Section 4 discusses the results of the proposed methods.

## 2. Materials and Method

### 2.1. GPS L1C Signal

Consider the GPS L1C received signal r(t) in Equations (1) and (2) [21]:

(1) $$r\left(t\right)=S_{L1C}\left(t\right)\cos\left(2\pi \left(f_{IF}+f_{D}\right)t\right)$$

(2) $$\mathrm{and}\;S_{L1C\;}\left(t\right)=\sqrt{\frac{3}{2}c}\cdot C_{OL}\left(t\right)C_{P}\left(t\right)SC_{P}\left(t\right)+\sqrt{\frac{c}{2}}\cdot D\left(t\right)C_{D}\left(t\right)SC_{D}\left(t\right)$$

where, the total signal power is denoted as *c* (Watts) which includes any antenna gain and receiver losses, $f_{IF}$ is the intermediate frequency, $f_{D}$ is the Doppler frequency, $C_{OL}\left(t\right)$ is the overlay code of the pilot channel, D(t) is the data, $C_{P}\left(t\right)\;\mathrm{and}\;C_{D}\left(t\right)$ are ranging codes of the pilot and data channel, respectively, and $SC_{P}\left(t\right)$ and $SC_{D}\left(t\right)$ are subcarriers of the pilot and data channel, respectively. The chip rate of the ranging codes, $C_{D}\left(t\right)\;\;\mathrm{and}\;C_{P}\left(t\right),$ is $f_{0}=1.023\;\mathrm{MHz},$ and the length of $C_{P}\left(t\right)\;\mathrm{and}\;C_{D}\left(t\right)$ is 10,230 chips (10 ms). The duration of each data bit and each chip of the overlay code is also 10 ms. The overlay code $C_{OL}\left(t\right)$ is 18 s in length at a rate of 100 bps, which results in the total length of 1800 bits.

**Figure 1:**
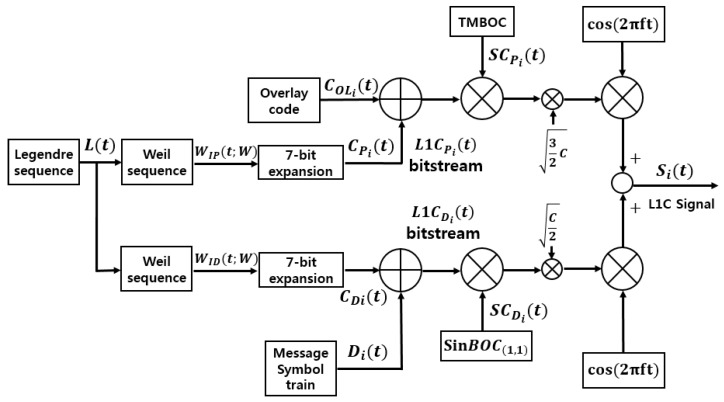
GPS L1C signal structure.

Figure 1 shows a generic view of the L1C signal generation, where the subscript *i* denotes the number of a satellite, but it is not used in the equations for the brevity of notation.

**Figure 2:**
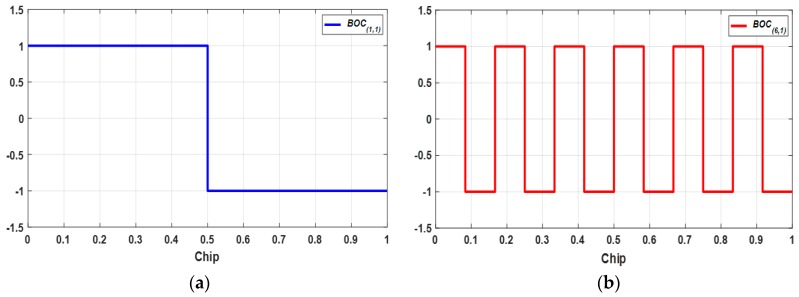
BOC modulation; (**a**) *BOC*_(1,1)_; (**b**) *BOC*_(6,1)_.

**Figure 3:**
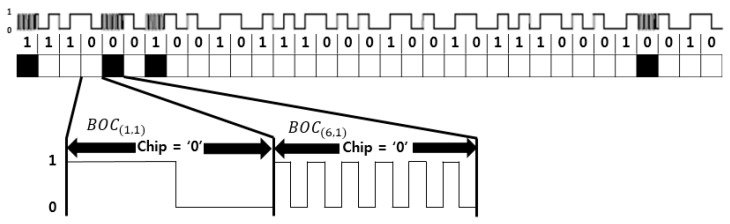
Structure of $TMBOC_{\left(6,1,\frac{4}{33}\right)}$.

Figure 1 shows the generation procedure of $C_{P}\left(t\right)\;\;\mathrm{and}\;C_{D}\left(t\right)$, while [6] gives the details. Equation (3) expresses the subcarriers $SC_{D}\left(t\right)$ and $SC_{P}\left(t\right)$, while Figure 2 and Figure 3 show the structure of *BOC*_(1,1)_, *BOC*_(6,1)_, and *TMBOC*. The *TMBOC* uses a time multiplexed combination of *BOC*_(1,1)_ and *BOC*_(6,1)_ as in Figure 3, where *BOC*_(6,1)_ is placed at the 0th, 4th, 6th, and 29th chip, and *BOC*_(1,1)_ is placed at the other chips among 33-chips. This pattern repeats 310 times, which makes 10,230 chips, and 10 ms in time:

(3) $${\mathrm{SC}}_{D}\left(t\right)=\sin BOC_{\left(1,1\right)}\left(t\right),\;{\mathrm{SC}}_{P}\left(t\right)=TMBOC_{\left(6,1,\frac{4}{33}\right)}\left(t\right)\;\;$$

where, $BOC_{\left(m,1\right)}\left(t\right)=\sin BOC_{\left(m,1\right)}\left(t\right)=\mathrm{sgn}\left(\sin\left(2\pi mf_{0}t\right)\right).$

In the notation of $TMBOC_{\left(6,1,\frac{4}{33}\right)},$ (6,1) stands for $BOC_{\left(6,1\right)}$ and $\frac{4}{33}$ means that $BOC_{\left(6,1\right)}$ is placed at 4 pre-designated chips in the 33-chip period as in Figure 3.

### 2.2. GPS L1C DM Signal

The received signal $r_{n}\left(t\right)$ of the GPS L1C signal with an additive Gaussian noise n(t) can be expressed as Equation (4):

(4) $$r_{n}\left(t\right)=S_{L1C}\left(t\right)\cos\left(2\pi \left(f_{IF}+f_{D}\right)t\right)+n\left(t\right),\;\;\;\;\mathrm{with}\;\;\;n\left(t\right)\sim N(0,\sigma_{n}^{2})$$

The DM (delay-and-multiply) signal of the received signal $r_{n}\left(t\right)$ is given with the delay δ as Equation (5):

(5) $$\begin{array}{lll} r_{\mathrm{DM},\delta }\left(t\right) & = & r_{n}\left(t\right)\cdot r_{n}\left(t-\delta \right)\\ & = & S_{L1C}\left(t\right)S_{L1C}\left(t-\delta \right)\cos\left(2\pi \left(f_{\mathrm{IF}}+f_{D}\right)t\right))\cos\left(2\pi \left(f_{\mathrm{IF}}+f_{D}\right)\left(t-\delta \right)\right)\\ & & +S_{L1C}\left(t\right)\cos\left(2\pi \left(f_{\mathrm{IF}}+f_{D}\right)t\right)n\left(t-\delta \right)\\ & & +S_{L1C}\left(t-\delta \right)\cos\left(2\pi \left(f_{\mathrm{IF}}+f_{D}\right)\left(t-\delta \right)\right)n\left(t\right)\\ & & +n\left(t\right)n\left(t-\delta \right) \end{array}$$

(6) $$=s_{DM,\;\delta }\left(t\right)+\eta_{DM,\delta }\left(t\right)$$

For the unit of delay δ, the chip of the ranging codes $C_{D}\left(t\right)\;\mathrm{and}\;C_{P}\left(t\right)$ can be used as δ = *n* chip, or the second can be used as $\delta =nT_{0}$ second, where $T_{0}=\frac{1}{f_{0}}=\frac{1}{1.023\times 10^{6}}\;s$.

The first term $s_{\mathrm{DM},\delta }\left(t\right)$ in Equation (5) is deterministic, and the following three terms, which are gathered in $\eta_{DM,\delta }\left(t\right),$ are random. The first term $s_{\mathrm{DM},\delta }\left(t\right)$ can be expressed as Equation (7), considering the removal of the high frequency term during the correlation process:

(7) $$s_{DM,\delta }\left(t\right)=r\left(t\right)r\left(t-\delta \right)\approx S_{L1C}\left(t\right)S_{L1C}\left(t-\delta \right)\times \frac{1}{2}\cos\left(2\pi \left(f_{IF}+f_{D}\right)\delta \right)$$

$$\begin{array}{ll} = & \frac{1}{2}\cos\left(2\pi \left(f_{IF}+f_{D}\right)\delta \right)\cdot \frac{c}{2}\left[\sqrt{3}C_{OL}\left(t\right)C_{P}\left(t\right)SC_{P}\left(t\right)+D\left(t\right)C_{D}\left(t\right)SC_{D}\left(t\right)\right]\\ & \cdot \left[\sqrt{3}C_{OL}\left(t-\delta \right)C_{P}\left(t-\delta \right)SC_{P}\left(t-\delta \right)+D\left(t-\delta \right)C_{D}\left(t-\delta \right)SC_{D}\left(t-\delta \right)\right]\\ = & d[3C_{OL}\left(t\right)C_{OL}\left(t-\delta \right)C_{P}\left(t\right)C_{P}\left(t-\delta \right)SC_{P}\left(t\right)SC_{P}\left(t-\delta \right)\\ & +\sqrt{3}\;D\left(t\right)C_{OL}\left(t-\delta \right)C_{D}\left(t\right)C_{P}\left(t-\delta \right)SC_{D}\left(t\right)SC_{P}\left(t-\delta \right)\\ & +\sqrt{3}C_{OL}\left(t\right)D\left(t-\delta \right)C_{P}\left(t\right)C_{D}\left(t-\delta \right)SC_{P}\left(t\right)\;SC_{D}\left(t-\delta \right)\\ & +D\left(t\right)D\left(t-\delta \right)C_{D}\left(t\right)C_{D}\left(t-\delta \right)SC_{D}\left(t\right)\;SC_{D}\left(t-\delta \right)] \end{array}$$

(8) $$\approx d[3C_{P}\left(t\right)C_{P}\left(t-\delta \right)SC_{P}\left(t\right)SC_{P}\left(t-\delta \right)\pm \sqrt{3}\;C_{D}\left(t\right)C_{P}\left(t-\delta \right)SC_{D}\left(t\right)SC_{P}\left(t-\delta \right)\;\;\pm \sqrt{3}C_{P}\left(t\right)C_{D}\left(t-\delta \right)SC_{P}\left(t\right)\;SC_{D}\left(t-\delta \right)+C_{D}\left(t\right)C_{D}\left(t-\delta \right)SC_{D}\left(t\right)\;SC_{D}\left(t-\delta \right)]$$

where:

(9) $$d=\frac{c}{4}\cos\left(2\pi \left(f_{IF}+f_{D}\right)\delta \right).$$

We used some approximation in Equation (8) such that $D\left(t\right)D\left(t-\delta \right)\approx 1,\;C_{OL}\left(t\right)C_{OL}\left(t-\delta \right)\approx 1,D\left(t\right)C_{OL}\left(t-\delta \right)\approx D\left(t-\delta \right)C_{OL}\left(t\right)\approx \pm 1$, where ≈ means that the term may have different values for only δ among 10,130 chips.

The term $\eta_{DM,\delta }\left(t\right)$ in Equation (6) consists of three noise terms as follows:

$$\eta_{DM,\delta }\left(t\right)=S_{L1C}\left(t\right)\cos\left(2\pi \left(f_{IF}+f_{D}\right)t\right)n\left(t-\delta \right)+S_{L1C}\left(t-\delta \right)\cos\left(2\pi \left(f_{\mathrm{IF}}+f_{D}\right)\left(t-\delta \right)\right)n\left(t\right)+n\left(t\right)n\left(t-\delta \right)$$

where the first and second terms are zero-mean random noise, and the third one is the product of two Gaussian noises. The product of two independent Gaussian random variables x and y with zero mean and variances $\sigma_{x}^{2}$ and $\sigma_{y}^{2}$ has a normal product distribution, as follows:

$$p_{xy}\left(u\right)=\int_{-\infty }^{\infty }\int_{-\infty }^{\infty }\frac{e^{\frac{-x^{2}}{2\sigma_{x}^{2}}}}{\sigma_{x}\sqrt{2\pi }}\cdot \frac{e^{\frac{-y^{2}}{2\sigma_{y}^{2}}}}{\sigma_{y}\sqrt{2\pi }}\cdot \delta \left(xy-u\right)dxdy=\frac{K_{0}\left(\frac{\left|u\right|}{\sigma_{x}\sigma_{y}}\right)}{\pi \sigma_{x}\sigma_{y}}$$

where, δ(x) is the Dirac delta function, and $K_{0}\left(x\right)$ is the modified Bessel function of the second kind and zero order, and the solution is $K_{0}\left(x\right)=\int_{0}^{\infty }\frac{\cos\left(xt\right)}{\sqrt{t^{2}+1}}dt$ [19].

## 3. Results

### 3.1. New DM Acquisition Schemes for GPS L1C Signal and the Unambiguous Property

#### 3.1.1. Choosing the Optimal Delay $\delta_{0}$ for the GPS DM Signal

The signal $S_{DM,\delta \;}\left(t\right)$ in Equation (8) can be expressed as Equation (10) using some simple notations as Equation (11):

(10) $$s_{DM,\delta \;}\left(t\right)=d[3C_{PP,\delta }\left(t\right)SC_{P}\left(t\right)SC_{P}\left(t-\delta \right)\pm \sqrt{3}\;C_{DP,\delta }\left(t\right)SC_{D}\left(t\right)SC_{P}\left(t-\delta \right)\;\pm \sqrt{3}C_{PD,\delta }\left(t\right)SC_{P}\left(t\right)\;SC_{D}\left(t-\delta \right)+C_{DD,\delta }\left(t\right)SC_{D}\left(t\right)\;SC_{D}\left(t-\delta \right)]$$

where:

$$C_{PP,\delta }\left(t\right)=C_{P}\left(t\right)C_{P}\left(t-\delta \right),\;\;\;\;\;\;\;C_{DP,\delta }\left(t\right)=C_{D}\left(t\right)C_{P}\left(t-\delta \right),$$

(11) $$C_{PD,\delta }\left(t\right)=C_{P}\left(t\right)C_{D}\left(t-\delta \right),\;\;\;\;\;\;\;\;C_{DD,\delta }\left(t\right)=C_{D}\left(t\right)C_{D}\left(t-\delta \right).$$

Equation (10) has two ± symbols, and the same sign occurs simultaneously, that is, (+) (+) or (−) (−), since $D\left(t\right)C_{OL}\left(t-\delta \right)\approx \;D\left(t-\delta \right)C_{OL}\left(t\right).$

**Figure 4:**
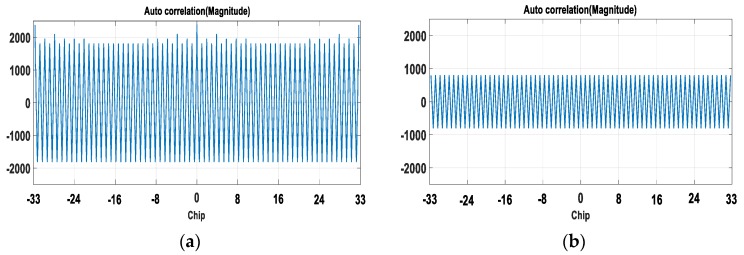
Auto correlation function (ACF) of; (**a**) $\sqrt{3}SC_{P}\left(t\right);$ (**b**) $SC_{D}\left(t\right)$.

Figure 4a is the auto correlation function (ACF) of $\sqrt{3}SC_{P}\left(t\right)$, which shows that it has peaks at δ= 33*n*-chips. Figure 4b shows the ACF of $SC_{D}\left(t\right)$, and it has peaks at every δ=0.5*n*-chip. The cross correlation function (CCF) of $\sqrt{3}SC_{P}\left(t\right)$ and $SC_{D}\left(t\right)$ shows a similar result to Figure 4b, and it also has peaks at every δ=0.5*n*-chip. Thus $\delta_{0}=$ 33*n-*chip will be candidate for the optimal delay.

Considering the condition of $\cos\left(2\pi f_{IF}\delta_{0}\right)=\pm 1$, the optimal delay $\delta_{0}$ in the GPS L1C DM signal is chosen as Equation (12):

(12) $$\delta_{0}=\{\begin{array}{cc} 33T_{0}, & n=1\\ \frac{k\pi }{2\pi f_{IF}}, & k=1,2,3,\;\cdots \end{array}$$

The optimal delay $\delta_{0}$ in Equation (12) is long compared with the Galileo case [18,19,20] and thus the Doppler frequency effect cannot be ignored in Equation (9). The value d becomes $d_{0}$ with condition (12):

(13) $$d_{0}=\frac{c}{4}\cos\left(2\pi \left(f_{IF}+f_{D}\right)\delta_{0}\right)=\frac{c}{4}\cos\left(k\pi +2\pi f_{D}\delta_{0}\right)=\pm \frac{c}{4}\cos\left(2\pi f_{D}\delta_{0}\right).$$

When $\cos\left(2\pi f_{D}\delta_{0}\right)=0.5$, we obtain that $f_{D}=5167\;\mathrm{Hz},$ which is the maximum Doppler frequency when a GPS receiver moves at the speed of 198 km/h. This calculation comes from the assumption of a circular orbit of the GPS satellite [3]. As is well-known, the Doppler frequency is slowly varying and the average rate of change of the Doppler frequency is 0.54 Hz/s for the stationary receiver [3], which is 0.567 Hz/s at the speed of 198 km/h. Thus $d_{0}$ in Equation (13) depends on the Doppler frequency, but can be treated as a constant during the acquisition process since the coherent integration time $T_{c}=10\;\mathrm{ms}$ for the GPS L1C signal.

The limitation that the proposed DM method which will be given in the following section can be used only for low speed due to the Doppler frequency can be solved by using combined L1 and L1C signal acquisition, which will be given in Section 3.4.

#### 3.1.2. The Proposed 33-chip Delay DM Acquisition Scheme

**Figure 5:**
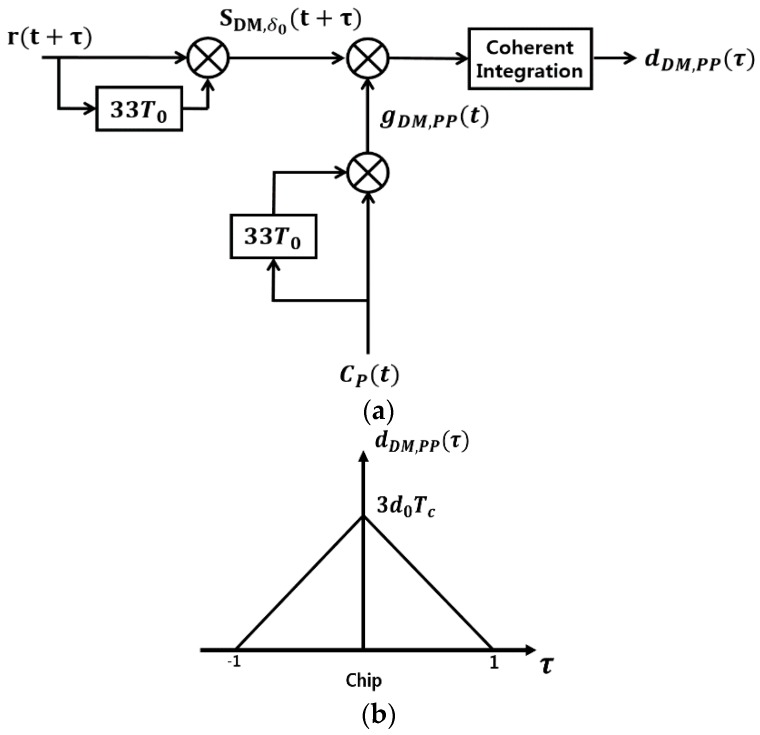
The proposed 33-chip delay DM acquisition scheme; (**a**) Block diagram; (**b**) The decision variable.

This section provides the two proposed DM acquisition schemes. Figure 5a shows the first 33-chip delay ($\delta_{0}=33T_{0}$) DM acquisition scheme.

The following Lemma shows the unambiguous property of the proposed 33-chip delay DM acquisition scheme in Figure 5a.

**Lemma** **1.** *Consider the GPS L1C DM signal $s_{DM,\delta_{0}}\left(t\right)$ in Equation (10) $\left(\delta =\delta_{0}=33T_{0}\right),$ and also the generated signal:*

$$g_{DM,PP}\left(t\right)=C_{PP,\delta_{0}}\left(t\right)=C_{P}\left(t\right)C_{P}\left(t-33T_{0}\right)$$

*then the CCF of $s_{DM,\delta_{0}}\left(t+\tau \right)$ and $g_{DM,PP}\left(t\right)$ during the coherent integration time $T_{c}$ = 10 ms is defined as the decision variable $d_{DM,PP}\left(\tau \right)=\int_{0}^{T_{c}}s_{DM,\delta_{0}}\left(t+\tau \right)g_{DM,PP}\left(t\right)dt$ in Figure 5a. The CCF can be obtained as:*

(14) $$d_{DM,PP}\left(\tau \right)=3d_{0}\left(1-\left|\tau \right|\right)\left(u\left(\tau +1\right)-u\left(\tau -1\right)\right)$$

*where $d_{0}=\pm \frac{c}{4}\cos\left(2\pi f_{D}\delta_{0}\right)$ and u(τ) is the unit step function.*

**Proof.**  Considering the optimal time delay $\sigma_{0}=33T_{0}$, we obtain:

$$SC_{P}\left(t\right)\cdot SC_{P}\left(t-\delta_{0}\right)=SC_{P}^{2}\left(t\right)=1,\;SC_{D}\left(t\right)\cdot SC_{D}\left(t-\delta_{0}\right)=SC_{D}^{2}\left(t\right)=1,\;\mathrm{and}$$

$$\int_{0}^{T_{c}}\;C_{DP,\delta_{0}}\left(t+\tau \right)C_{PP,\delta_{0}}\left(t\right)dt\;\approx 0,\;\int_{0}^{T_{c}}\;C_{PD,\delta_{0}}\left(t+\tau \right)C_{PP,\delta_{0}}\left(t\right)dt\approx 0,\;\int_{0}^{T_{c}}\;C_{DD,\delta_{0}}\left(t+\tau \right)C_{PP,\delta_{0}}\left(t\right)dt\approx 0.$$

$$\begin{array}{ll} d_{DM,PP}\left(\tau \right) & =\int_{0}^{T_{c}}s_{DM,\delta_{0}}\left(t+\tau \right)g_{DM,PP}\left(t\right)dt\\ & =\int_{0}^{T_{c}}d[3C_{PP,\delta_{0}}\left(t+\tau \right)\pm \sqrt{3}\;C_{DP,\delta_{0}}\left(t+\tau \right)SC_{D}\left(t+\tau \right)SC_{P}\left(t+\tau -\delta_{0}\right)\;\\ & \pm \sqrt{3}C_{PD,\delta_{0}}\left(t\right)SC_{P}\left(t+\tau \right)\;SC_{D}\left(t+\tau -\delta_{0}\right)+C_{DD,\delta_{0}}\left(t+\tau \right)]C_{PP,\delta_{0}}\left(t\right)d\\ & \approx 3d_{0}\int_{0}^{T_{c}}C_{PP,\delta_{0}}\left(t+\tau \right)C_{PP,\delta_{0}}\left(t\right)dt \end{array}$$

Since $C_{PP,\delta_{0}}\left(t\right)$ is a pseudo random noise (PRN) signal, the $d_{DM,PP}\left(\tau \right)$ is the 3$d_{0}$ times of ACF of $C_{PP,\delta_{0}}\left(t\right)$, which has the form of Equation (14), and is shown as Figure 5b.□

**Figure 6:**
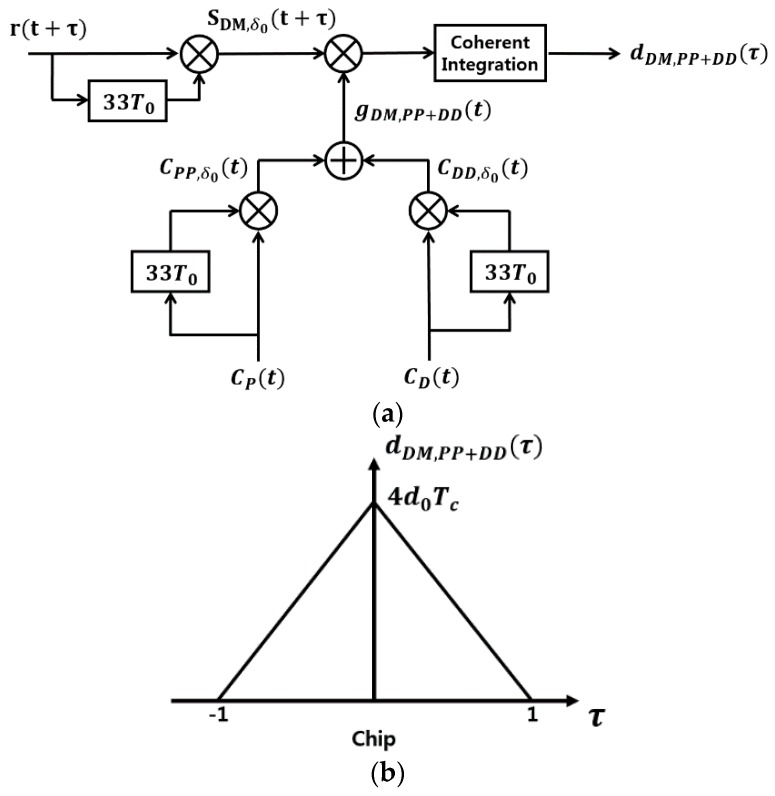
The proposed 33-chip delay DM acquisition scheme; (**a**) Block diagram; (**b**) The decision variable.

Figure 6a shows the second 33-chip delay ($\delta_{0}=33T_{0}$) DM acquisition scheme.

The following Lemma shows another 33-chip delay DM acquisition scheme as in Figure 6a.

**Lemma** **2.** *Consider the GPS L1C DM signal $s_{DM,33T_{0}}\left(t\right)$ in Equation (10) $\left(\delta =\delta_{0}=33T_{0}\right)$ and also the generated signal:*

(15) $$g_{DM,PP+DD}\left(t\right)=C_{PP,\delta_{0}}\left(t\right)+C_{DD,\delta_{0}}\left(t\right)=\;\;C_{P}\left(t\right)C_{P}\left(t-33T_{0}\right)+C_{D}\left(t\right)C_{D}\left(t-33T_{0}\right).$$

*then the CCF of $s_{DM,\delta_{0}}\left(t+\tau \right)$ and $g_{DM,PP+DD}\left(t\right)$ during the coherent integration time $T_{c}$ = 10 ms is defined as the decision variable $d_{DM,PP+DD}\left(\tau \right)=\int_{0}^{T}s_{DM,\delta_{0}}\left(t+\tau \right)g_{DM,PP+DD}\left(t\right)dt$ in Figure 6a. The CCF can be obtained as:*

(16) $$d_{DM,PP+DD}\left(\tau \right)=4d_{0}\left(1-\left|\tau \right|\right)\left(u\left(\tau +1\right)-u\left(\tau -1\right)\right)$$

*where $d_{0}=\pm \frac{c}{4}cos\left(2\pi f_{D}\delta_{0}\right)$ and u(τ) is the unit step function.*

**Proof.**  Using the similar manipulation as in the Proof of Lemma 1, we obtain that:

(17) $$\begin{array}{ll} d_{DM,PP+DD}\left(\tau \right) & =\int_{0}^{T_{c}}s_{DM,\delta_{0}}\left(t+\tau \right)g_{DM,PP+DD}\left(t\right)dt\\ & =\int_{0}^{T_{c}}d_{0}[3C_{PP,\delta_{0}}\left(t+\tau \right)\pm \sqrt{3}\;C_{DP,\delta_{0}}\left(t+\tau \right)SC_{D}\left(t\right)SC_{P}\left(t+\tau -\delta_{0}\right)\\ & \pm \sqrt{3}C_{PD,\delta_{0}}\left(t+\tau \right)SC_{P}\left(t\right)\;SC_{D}\left(t+\tau -\delta_{0}\right)+C_{DD,\delta_{0}}\left(t+\tau \right)]\cdot \{C_{PP,\delta_{0}}\left(t\right)+C_{DD,\delta_{0}}\left(t\right)\}dt\\ & \approx 3d_{0}\int_{0}^{T_{c}}C_{PP,\delta_{0}}\left(t+\tau \right)C_{PP,\delta_{0}}\left(t\right)dt\;+\;d_{0}\int_{0}^{T_{c}}C_{DD,\delta_{0}}\left(t+\tau \right)C_{DD,\delta_{0}}\left(t\right)dt\\ & =4d_{0}\left(1-\left|\tau \right|\right)\left(u\left(\tau +1\right)-u\left(\tau -1\right)\right) \end{array}$$

Since $C_{PP,\delta_{0}}\left(t\right)$ and $C_{DD,\delta_{0}}\left(t\right)$ are PRN signals, the $d_{DM,PP+DD}\left(\tau \right)$ is the ACF of $C_{PP,\delta_{0}}\left(t\right)$ plus the ACF of $C_{PP,\delta_{0}}\left(t\right)$, which has the form of Equation (16), and is shown as Figure 6b.□

#### 3.1.3. Determination of the Doppler Frequency

In this section we show the procedure to find the Doppler frequency of the received signal $r_{n}\left(t\right)$ after we obtain the code phase through the proposed DM acquisition scheme.

**Figure 7:**
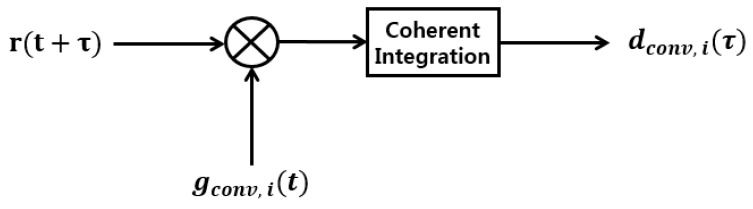
The conventional acquisition scheme.

Consider the received signal $r_{n}\left(t\right)$ and the generated signal $g_{conv,P}\left(t\right)$ in Figure 7 with τ=0. Then:

(18) $$\begin{array}{ll} r_{n}\left(t\right)\cdot g_{conv,P}\left(t\right) & =\left[\left\{\sqrt{\frac{3}{2}c}\cdot C_{OL}\left(t\right)C_{P}\left(t\right)SC_{P}\left(t\right)+\sqrt{\frac{c}{2}}\cdot D\left(t\right)C_{D}\left(t\right)SC_{D}\left(t\right)\right\}\cos\left(2\pi \left(f_{IF}+f_{D}\right)t\right)+n\left(t\right)\right]C_{P}\left(t\right)SC_{P}\left(t\right)\cos\left(2\pi f_{IF}t\right)\\ & =\left\{\pm \sqrt{\frac{3}{2}c}\pm \sqrt{\frac{c}{2}}C_{D}\left(t\right)C_{P}\left(t\right)SC_{D}\left(t\right)SC_{P}\left(t\right)\right\}\cdot \frac{1}{2}(\cos\left(2\pi f_{D}t\right)+\cos\left(2\pi \left(2f_{IF}+f_{D}\right)t\right)\\ & \;\;\;+n\left(t\right)\cdot C_{P}\left(t\right)SC_{P}\left(t\right)\cos\left(2\pi f_{IF}t\right)\\ & \approx \pm \frac{1}{2}\sqrt{\frac{3}{2}c}\cdot \cos\left(2\pi f_{D}t\right) \end{array}$$

where we use that $C_{P}^{2}\left(t\right)=1,\;SC_{P}^{2}\left(t\right)=1,$ and the ranging code and the Gaussian noise term will be filtered out.

By performing the fast Fourier transform for the signal (18) we can obtain the Doppler frequency $f_{D}$ with Lemma 3.

**Lemma** **3.** *Suppose that a continuous-time signal* x(t) *is a sinusoidal signal with frequency* $f_{D}$*. Define* $f_{s}$ *as the sampling frequency*, *and* T *as the sampling interval in the time domain.*

*The sampled sequence* x[n],  1≤n≤M, *has a discrete-time Fourier transform (DFT)* X(k), 1≤k≤M*:*

*(1)*  *Then the resolution of* X(k) *is* $\Delta f=\frac{1}{T}\;\left[Hz\right].$
*(2)*  *If we reduce the number of samples by averaging* x[n] *while maintaining the Nyquist theorem such that* $x\left[\bar{n}\right],\;\;1\leq \bar{n}\leq \bar{M},\;\;M=L\bar{M},$ *and the DFT is* $X\left(\bar{k}\right),\;\;1\leq \bar{k}\leq \bar{M},$ *then the resolution of* $X\left(\bar{k}\right)$ *is still* $\Delta f=\frac{1}{T}\;\left[Hz\right].$

**Proof.**  (1) From the sampling frequency and the sampling interval, the number of samples is $M=f_{s}\cdot T.$ It is well-known that the highest frequency of the sampled data is $\frac{f_{s}}{2}$ and the highest frequency of X(k) is located at $\frac{M}{2}$ (suppose that M is even). There ore e obtain $\Delta f\cdot \frac{M}{2}=\frac{f_{s}}{2}=\frac{1}{2}\frac{M}{T},$ which results in $\Delta f=\frac{1}{T}\;\left[\mathrm{Hz}\right].$ (2) From the new sampling frequency $\bar{f_{s}}=\frac{f_{s}}{L}$ and the number of samples $\bar{M}=\frac{M}{L}$, the similar Equation holds as in proof (1) such as $\Delta f\cdot \frac{\bar{N}}{2}=\frac{\bar{f_{s}}}{2}=\frac{1}{2}\frac{\bar{N}}{T},$ which results in the same Equation, $\Delta f=\frac{1}{T}\;\left[\mathrm{Hz}\right].$ □

Considering (18), the above method cannot distinguish positive Doppler frequency from negative Doppler frequency since $\cos\left(2\pi f_{D}t\right)=\cos\left(2\pi \left(-f_{D}\right)t\right).$ Thus after finding the Doppler frequency using Lemma 3, the rate of the relative velocity of the vehicle from the satellite should be used to determine the sign of the Doppler frequency.

### 3.2. Noise Characteristics of the Proposed DM and Conventional Acquisition Method

#### 3.2.1. Noise Characteristics of the Proposed DM Acquisition Method

In this section, we show the SNR of the decision variables for the proposed DM acquisition methods, which are shown in Figure 5a and Figure 6a.

**Lemma** **4.** *Consider the proposed DM acquisition scheme in Figure 5a, where the generated signal is* $g_{DM,PP}\left(t\right)=C_{PP,\delta_{0}}\left(t\right)=C_{P}\left(t\right)C_{P}\left(t-33T_{0}\right).$ *The SNR of the decision variable in the presence of GPS L1C signal can be approximated as Equation (19):*

(19) $$SNR_{DM,PP}=\frac{\frac{9}{16}\cdot c^{2}{\left\{\cos\left(2\pi f_{D}\delta_{0}\right)\right\}}^{2}M}{\sigma_{n}^{4}+2c\cdot \sigma_{n}^{2}}$$

*where* $\delta_{0}=33T_{0}$ *is the optimal delay in DM signal,* M *is the number of samples according to the appropriate sampling frequency and the coherent integration period* $T_{c},$ *and* $\sigma_{n}^{2}$ *is the variance of the noise* n(t) *in Equation (4).*

**Proof.**  Denote $H_{0}$ as the hypothesis in the absence of GPS L1C signal, and $H_{1}$ in the presence of the signal, and $Z_{H_{0}}$ and $Z_{H_{1}}$ as the decision variables in the case of $H_{0}$ and $H_{1}$, respectively. Suppose that the optimal time delay is $\delta_{0}=33T_{0},$ and the generated signal is $g_{DM,PP}\left(t\right)=C_{PP,\delta_{0}}\left(t\right)=C_{P}\left(t\right)C_{P}\left(t-33T_{0}\right)$, then we obtain the decision variables for $H_{0}$ and $H_{1}$ as follows:

$$H_{0}\;:Z_{H_{0}}=\overset{M-1}{\underset{i=0}{\sum }}n\left(t_{i}\right)n\left(t_{i}-33T_{0}\right)C_{PP,\delta_{0}}\left(t\right)$$

Denote $n_{DM,\delta_{0}}\left(t\right)=n\left(t\right)n\left(t-33T_{0}\right),$ then it is known that $E\left[n_{DM,\delta_{0}}\right]=0,\;\sigma_{n_{DM,\delta_{0}}}^{2}=\sigma_{n}^{4}$ [19].

The signal $n_{DM,\delta_{0}}\left(t\right)$ is actually a random process, and thus $n_{DM,\delta_{0}}\left(t_{i}\right),\;i=0,\;1,2,\cdots ,\;M-1,$ are random variables that are independent and identically distributed. Thus by the central limit theorem, $Z_{H_{0}}$ has the Gaussian distribution with mean zero and variance, $M\sigma_{n}{}^{4}$, that is:

$$H_{0}:\;\;\;\;\;Z_{H_{0}}\;\sim \;N\left(0,\;M\sigma_{n}{}^{4}\right)$$

Suppose that the GPS L1C signal is present, and that the received signal and the generated signal are aligned in phase, then the decision variable becomes:

(20) $$\begin{array}{l} H_{1}\;\;:\;Z_{H_{1}}=\overset{M-1}{\underset{i=0}{\sum }}r_{DM,\delta_{0}}\left(t_{i}\right)C_{PP,\delta_{0}}\left(t_{i}\right)\\ =\overset{M-1}{\underset{i=0}{\sum }}s_{DM,\delta_{0}}\left(t_{i}\right)\;C_{PP,\delta_{0}}\left(t_{i}\right)+\overset{M-1}{\underset{i=0}{\sum }}S_{L1C}\left(t_{i}\right)\cos\left(2\pi \left(f_{IF}+f_{D}\right)t_{i}\right)\;n\left(t_{i}-\delta_{0}\right)C_{PP,\delta_{0}}\left(t_{i}\right)\\ \;\;+\sum_{i=0}^{M-1}S_{L1C}\left(t_{i}-\delta_{0}\right)\cos\left(2\pi \left(f_{IF}+f_{D}\right)(t_{i}-\delta_{0}\right)\;n\left(t_{i}\right)C_{PP,\delta_{0}}\left(t_{i}\right)\\ \;\;+\sum_{i=0}^{M-1}n_{DM,\delta_{0}}\left(t_{i}\right)C_{PP,\delta_{0}}\left(t_{i}\right) \end{array}$$

The first term is given using Equation (14) as follows:

$$Z_{H_{1,1}}=\sum_{i=0}^{M-1}s_{DM,\delta_{0}}\left(t_{i}\right)\;C_{PP,\delta_{0}}\left(t_{i}\right)\approx 3d_{0}\sum_{i=0}^{M-1}C_{PP,\delta_{0}}\left(t_{i}\right)C_{PP,\delta_{0}}\left(t_{i}\right)=3d_{0}M=\frac{3c}{4}\cos\left(2\pi f_{D}\delta_{0}\right)M.$$

The second term of Equation (20) has the form of $Z_{H_{1,2}}=\sum_{i=0}^{M-1}\;a_{i}n\left(t_{i}-\delta_{0}\right),$ where $a_{i}=S_{L1C}\left(t_{i}\right)C_{PP,\delta_{0}}\left(t_{i}\right)\cos\left(2\pi \left(f_{IF}+f_{D}\right)t_{i}\right),$ is the linear combination of independent and identically distributed random variables. Thus by the central limit theorem, $Z_{H_{1,2}}$ has the Gaussian distribution with mean zero and variance $\sigma_{Z_{H_{1,2}}}^{2}$:

$$\begin{array}{ll} \sigma_{Z_{H_{1,2}}}^{2} & =\overset{M-1}{\underset{i=0}{\sum }}\;a_{i}^{2}\sigma_{n}^{2}=\overset{M-1}{\underset{i=0}{\sum }}\;\left\{S_{L1C}^{2}\left(t_{i}\right)\cos^{2}\left(2\pi \left(f_{IF}+f_{D}\right)t_{i}\right)C_{PP,\delta_{0}}^{2}\left(t_{i}\right)\right\}\sigma_{n}^{2}\\ & =\sigma_{n}^{2}\sum_{i=0}^{M-1}\;\left\{S_{L1C}^{2}\left(t_{i}\right)\cos^{2}\left(2\pi \left(f_{IF}+f_{D}\right)t_{i}\right)\right\}\\ & =\sigma_{n}^{2}\sum_{i=0}^{M-1}\;\left\{\frac{1}{2}\left\{\left(2+\sqrt{3}\right)c+\left(2-\sqrt{3}\right)c\right\}\cdot \frac{1-\cos\left(4\pi \left(f_{IF}+f_{D}\right)t_{i}\right)}{2}\right\}\approx cM\sigma_{n}^{2} \end{array}$$

where, we use the property that $S_{L1C}^{2}\left(t_{i}\right)$ has two values of $(2+\sqrt{3}$)*c* or $(2-\sqrt{3}$)*c* with equal opportunity, and the high frequency signal is filtered out.

Similarly, we obtain that $E\left[Z_{H_{1,3}}\right]=0,\sigma_{Z_{H_{1,3}}}^{2}\approx cM\sigma_{n}^{2}.$ Also $E\left[Z_{H_{1,4}}\right]=0,\;\sigma_{Z_{H_{1,4}}}^{2}=M\sigma_{n}^{4}.$

In summary, the decision variable has the Gaussian distribution as follows:

$$Z_{H_{1}}\sim N\left(\frac{3c}{4}\cos\left(2\pi f_{D}\delta_{0}\right)M,\;\;\left(\sigma_{n}^{4}+2c\sigma_{n}^{2}\right)M\right).$$

Thus the SNR is obtained as follows:

$$SNR_{DM,PP}=\frac{E{\left[Z_{H_{1}}\right]}^{2}}{\sigma_{H_{1}}^{2}}=\frac{{\left(\frac{3}{4}c\cdot \cos\left(2\pi f_{D}\delta_{0}\right)M\right)}^{2}}{\left(\sigma_{n}^{4}+2c\sigma_{n}^{2}\right)M}=\frac{\frac{9}{16}\cdot c^{2}\cos^{2}\left(2\pi f_{D}\delta_{0}\right)M}{\sigma_{n}^{4}+2c\sigma_{n}^{2}}$$

□

**Lemma** **5.** *Consider the proposed DM acquisition scheme in Figure 6a, where the generated signal is* $g_{DM,PP+DD}\left(t\right)=C_{PP,\delta_{0}}\left(t\right)+C_{DD,\delta_{0}}\left(t\right)=C_{P}\left(t\right)C_{P}\left(t-33T_{0}\right)+C_{D}\left(t\right)C_{D}\left(t-33T_{0}\right).$ *The SNR of the decision variable in the presence of GPS L1C signal can be approximated as Equation (21):*

(21) $$SNR_{DM,PP+DD}=\frac{\frac{1}{2}\cdot c^{2}{\left\{\cos\left(2\pi f_{D}\delta_{0}\right)\right\}}^{2}M}{\sigma_{n}^{4}+2c\cdot \sigma_{n}^{2}}$$

*where* $\delta_{0}=33T_{0}$ *is the optimal delay in DM signal,* M *is the number of samples according to the sampling frequency and the coherent integration period, and* $\sigma_{n}^{2}$ *is the variance of the noise* n(t) *in Equation (4).*

The proof of Lemma 5 has similar procedure as that of Lemma 4, and thus is omitted here and is given in Appendix A.

#### 3.2.2. Noise Characteristics of the Conventional Acquisition Method

In this section, we show the SNR of the decision variables for the possible conventional acquisition methods which are shown in Figure 7.

**Lemma** **6.** *Consider the conventional acquisition scheme in Figure 7, and assume that we know the Doppler frequency, and thus we use f_D_ = 0. Note that the generated signal is $g_{conv,i}\left(t\right)$. We consider three cases of generated signals, which are:*

$$\begin{array}{l} g_{conv,P}\left(t\right)=C_{P}\left(t\right)SC_{P}\left(t\right)\cos\left(2\pi f_{IF}t\right)\\ g_{conv,\sqrt{3}P+D}\left(t\right)=\left\{\sqrt{3}C_{P}\left(t\right)SC_{P}\left(t\right)+C_{D}\left(t\right)SC_{D}\left(t\right)\right\}\cos\left(2\pi f_{IF}t\right)\\ g_{conv,P+D}\left(t\right)=\left\{C_{P}\left(t\right)SC_{P}\left(t\right)+C_{D}\left(t\right)SC_{D}\left(t\right)\right\}\cos\left(2\pi f_{IF}t\right) \end{array}$$

The SNR of the decision variable in the presence of the GPS L1C signal can be approximated for the three cases as (22):

(22) $$SNR_{conv,P}=\frac{\frac{3}{4}c\cdot M}{\sigma_{n}^{2}},\;SNR_{conv,\sqrt{3}P+D}=\frac{\frac{1}{4}c\cdot M}{\sigma_{n}^{2}},\;\;\;\;\;\;\;SNR_{conv,P+D}=\frac{\frac{1}{2}c\cdot M}{\sigma_{n}^{2}}\;\mathrm{or}\;0$$

*where*,  M *is the number of samples according to the sampling frequency and the coherent integration period, and* $\sigma_{n}^{2}$ *is the variance of the noise* n(t) *in Equation (4).*

The proof of Lemma 6 has similar procedure as that of Lemma 4, and thus is omitted here and given in Appendix B.

#### 3.2.3. Comparison of the SNR Performance between DM and Conventional Methods

This section summarizes the SNR ratio in the presence of L1C signal for the DM and conventional methods from the results of Lemmas 4–6. Table 1 compares the performance with each other. For the two DM methods, the SNR of $g_{DM,PP}$ is greater than that of $g_{DM,PP+DD}$, while the mean value, which is the correlation peak, of $g_{DM,PP+DD}$ is greater than that of $g_{DM,PP}.$ In this case the use of $g_{DM,PP}$ provides greater probability of detection than that of $g_{DM,PP+DD}$. Thus, we can conclude that the DM method of $g\left(t\right)=g_{DM,PP}\left(t\right)$ is preferable to obtain better performance such as the probability of detection. In similar way, $g\left(t\right)=g_{conv,P}\left(t\right)$ is the best among the three candidates in the conventional method.

As mentioned in Section 3.1.1, the optimal delay $\delta_{0}=33T_{0}$ is long and thus we cannot ignore the effect of the Doppler frequency such that $0.5\leq |\cos\left(2\pi f_{D}\delta_{0}\right)|\leq 1$, for $-5167\;\mathrm{Hz}\leq f_{D}\leq 5167\;\mathrm{Hz},$ which is the maximum Doppler frequency when a GPS receiver moves at the speed of 198 km/h. The Doppler frequency is slowly varying and thus the cosine value in Table 1 can be treated as a constant for the acquisition process. The following Corollary shows the ratio of SNR between the DM method and the conventional method.

**Corollary** **1.** *Consider the SNR of the decision variable for the DM acquisition method* $SNR_{DM,PP}$ *and the SNR of the decision variable for the conventional acquisition method* $SNR_{conv,P}$*. Then,* $SNR_{DM,PP}$ *is always less than* $SNR_{conv,P}$*, and the SNR ratio is given as Equation (23):*

(23) $$\frac{SNR_{DM,PP}}{SNR_{conv,P}}=\frac{\frac{3}{4}c\cdot \cos^{2}\left(2\pi f_{D}\delta_{0}\right)}{\sigma_{n}^{2}+2c}.$$

#### 3.2.4. Probability of Detection with Respect to the Number of Samples

In this section we consider the effect of the number of samples on the detection probability. It is well-known that when a longer data is used for the acquisition process, the SNR increases and the detection performance enhances. This performance improvement can be obtained through the fast sampling, which increases M in the DM method in Table 1

The following Lemma shows that when the sampling speed increases, meaning the increase of M, the detection probability increases.

**Lemma** **7.** *Consider the generated signal* $g\left(t\right)=g_{DM,PP}\left(t\right)$ *in the DM acquisition method in Table 1. Assuming that* c=1 *and* $\cos\left(2\pi f_{IF}\delta_{0}\right)\approx 1,$ *we obtain that* $Z_{H_{0}}\;\sim N\left(0,\;\;M\sigma_{n}^{4}\right)$ *and* $Z_{H_{1}}\;\sim N\left(\frac{3}{4}M,\;\;\left(\sigma_{n}^{4}+2\sigma_{n}^{2}\right)M\right),$ *where* M *is the number of samples according to the appropriate sampling frequency and the coherent integration period T. Suppose that the probability of false alarm is given, then the detection probability* $P_{d}$ *increases as the number of sample* M *increases, i.e.:*

$$if\;M_{1}<M_{2},\;\mathrm{then}\;P_{d}^{M_{1}}<P_{d}^{M_{2}}.$$

**Proof.**  Define the probability density functions of the decision variables $Z_{H_{0}}$ and $Z_{H_{1}}$ as $f_{Z_{H_{0}}}\left(z\right)$ and $f_{Z_{H_{1}}}\left(z\right)$. When the probability of false alarm $P_{fa}$ is given, the threshold γ and the probability of detection $P_{d}$ is given as follows: $\int_{-\infty }^{\gamma }f_{Z_{H_{0}}}\left(z\right)dz=P_{fa}$ and $\int_{\gamma }^{\infty }f_{Z_{H_{1}}}\left(z\right)dz=P_{d}$.

Consider two sample numbers $M_{1}$ and $M_{2}$ with $M_{1}<M_{2}$, and the corresponding thresholds $\gamma_{1}$ and $\gamma_{2}$, and the corresponding probability of detection, as $P_{d}^{M_{1}}$ and $P_{d}^{M_{2}}$. We will show that $\gamma_{1}<\gamma_{2}$ first and then $P_{d}^{M_{1}}<P_{d}^{M_{2}}$.

(i) Suppose that the probability of false alarm $P_{fa}$ is given. Despite of the change of variance, the normalized threshold does not change, i.e., $\frac{\gamma_{1}}{\sqrt{M_{1}}\sigma_{n}^{2}}=\frac{\gamma_{2}}{\sqrt{M_{2}}\sigma_{n}^{2}},$ which becomes $\frac{\gamma_{1}}{\gamma_{2}}=\sqrt{\frac{M_{1}}{M_{2}}}<1.$ Hence, we have $\gamma_{1}<\gamma_{2}.$
(ii) The probability of detection is given for the sample numbers $M_{1}$ and $M_{2}$ as:
  $$\int_{\gamma_{1}}^{\infty }f_{Z_{H_{1}}}^{M_{1}}\left(z\right)dz=P_{d}^{M_{1}}\;\mathrm{and}\;\int_{\gamma_{2}}^{\infty }f_{Z_{H_{2}}}^{M_{2}}\left(z\right)dz=P_{d}^{M_{2}}.$$
  The normalized thresholds are $\bar{\gamma_{1}}=\frac{\gamma_{1}-\frac{3}{4}M_{1}}{\sqrt{M_{1}}\cdot \sqrt{\sigma_{n}^{4}+2\sigma_{n}^{2}}}$ and $\bar{\gamma_{2}}=\frac{\gamma_{2}-\frac{3}{4}M_{2}}{\sqrt{M_{2}}\cdot \sqrt{\sigma_{n}^{4}+2\sigma_{n}^{2}}}$, which becomes:
  $$\frac{\bar{\gamma_{1}}}{\bar{\gamma_{2}}}=\sqrt{\frac{M_{2}}{M_{1}}}\cdot \frac{\gamma_{1}-\frac{3}{4}M_{1}}{\gamma_{2}-\frac{3}{4}M_{2}}=\sqrt{\frac{M_{2}}{M_{1}}}\cdot \frac{\gamma_{2}\cdot \sqrt{\frac{M_{1}}{M_{2}}}-\frac{3}{4}M_{1}}{\gamma_{2}-\frac{3}{4}M_{2}}=\frac{\gamma_{2}-\frac{3}{4}\sqrt{M_{1}M_{2}}}{\gamma_{2}-\frac{3}{4}M_{2}}>1.\;\mathrm{Thus}\;\bar{\gamma_{2}}<\bar{\gamma_{1}},\;\text{meaning that}\;P_{d}^{M_{1}}<P_{d}^{M_{2}}.$$

□

#### 3.2.5. Comparison of Search Time for Coarse Acquisition

This section compares the acquisition time taken roughly by both the conventional method and the proposed DM method. Suppose that the software receiver is used with the fast Fourier transform (FFT) method and the Doppler frequency search bin is 50 Hz for brevity. Then the conventional method should search the Doppler frequency 209 (= 10.4 kHz/50 Hz + 1) times. The Doppler frequency of 5.2 kHz comes from the discussion in Section 3.1.1. For the DM approach, the generated signal $g_{DM,PP}\left(t\right)$ in Figure 5a and $g_{DM,PP+DD}\left(t\right)$ in Figure 6a can be precalculated and stored in the memory. Thus only one more multiplication is necessary for the DM approach to obtain $s_{DM,\delta_{0}}\left(t\right)$ before correlation, compared with the conventional method in Figure 7. The correlation of the received signal $r_{DM}\left(t\right)$ and the generated signal $g_{DM}\left(t\right)$ is performed through the parallel code phase search acquisition using FFT [2] in this paper. However, we count it as 1 correlation for simplicity. The search time to obtain both code phase and Doppler frequency can be roughly described with the Doppler frequency resolution Δf=50 Hz as follows:

(1) The proposed DM approach:
  K *(1 multiplication + 1 correlation)         to search the code phase
  +1 multiplication + 1 FFT (much reduced order)  to obtain the Doppler frequency
  ≈(K + 1) multiplication + K correlations
(2) The conventional approach:
  209 correlations + find maximum peak ≈ 209 correlations

where K is the number of non-coherent integration.

Thus the search time of the DM acquisition method depends on K, which depends on the noise environment.

**Comment** **1.** According to Lemma 7, the probability of detection can be increased as the number of sample increases, which can be obtained by sampling more fast as well as by using longer samples like non-coherent integration. So the acquisition time of the DM approach can be reduced much with higher sampling rate due to the advance of sampling technology.

### 3.3. Results of the Performance Analysis for the Proposed DM Acquisition Method

In this section we simulate the acquisition process for both conventional and proposed DM methods. Assume that the intermediate frequency is $f_{IF}=6.2\;\mathrm{MHz}$, the sampling frequency is $f_{s}=60\;\mathrm{MHz},$ and the Doppler frequency $f_{D}=0$. The coherent integration time is $T_{c}$ = 10 ms and the number of samples is $M=f_{s}T_{c}$ = 600,000.

#### 3.3.1. The Correlation Function of DM and Conventional Method

**Figure 8:**
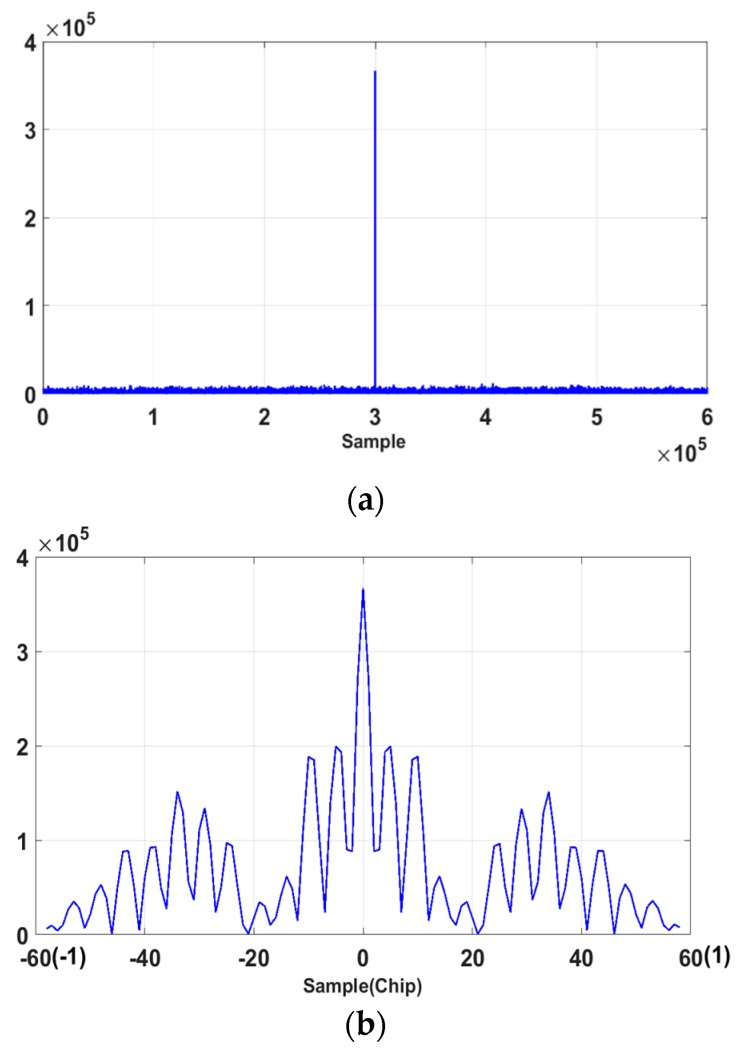
The CCF for the conventional method; (**a**) Correlation plot for 10,230 chips (600,000 samples); (**b**) Correlation plot for ±1 chip (59 samples) around the correlation peak.

The decision variable $d_{conv,P}\left(\tau \right)$ in Figure 7, which is the CCF of the received signal r(t+τ) and the generated signal $g\left(t\right)=\;g_{conv,P}\left(t\right)$ for the conventional acquisition scheme is plotted with respect to the code phase τ in Figure 8. 

The correlation peak is shown in Figure 8a and shown again in detail around the peak within ±1 chip $\left(T_{0}=\frac{1}{1.023\times 10^{6}}\;s\right)$ in Figure 8b, which shows severe multiple peaks around the maximum peak.

**Figure 9:**
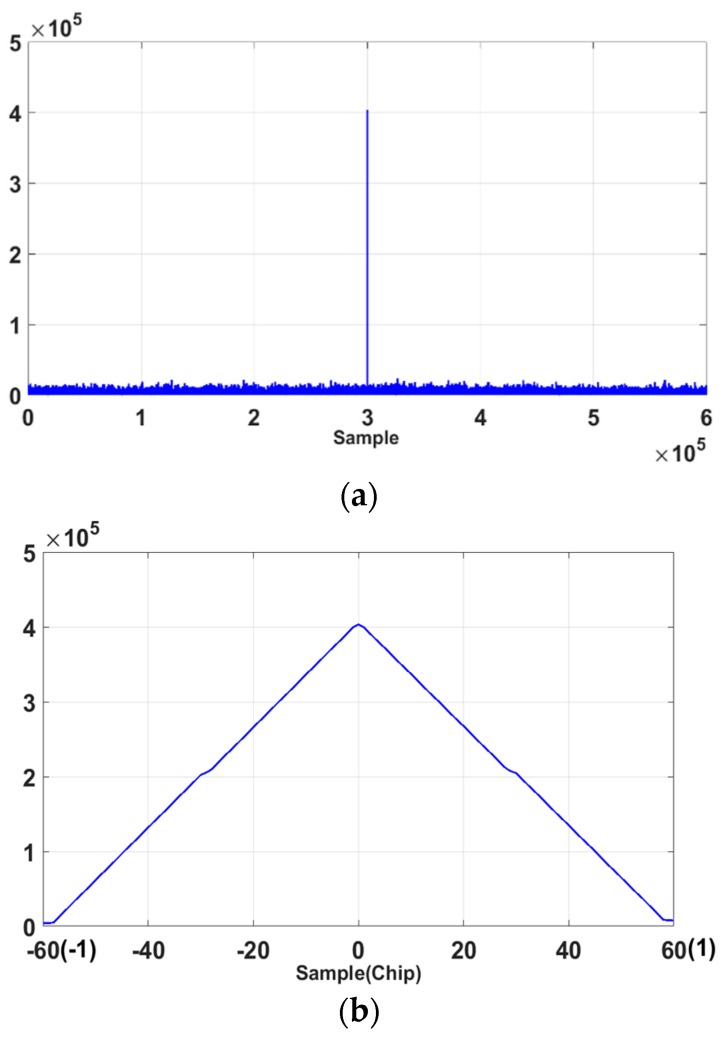
The CCF for the DM method (33-chip delay); (**a**) Correlation plot for 10,230 chips (600,000 samples); (**b**) Correlation plot for ±1 chip (59 samples) around the correlation peak.

The decision variable $d_{DM,PP}\left(\tau \right)$ in Figure 5 is the CCF of the received DM signal $s_{DM,\sigma_{0}}\left(t+\tau \right)$ and the generated DM signal $g\left(t\right)=g_{DM,PP}\left(t\right)$ for the DM acquisition scheme and is plotted with respect to the code phase τ in Figure 9. The correlation peak is shown in Figure 9a and shown again in detail around the peak within ±1 chip in Figure 8b, where we can see that the multiple peaks around the maximum peak are completely removed.

#### 3.3.2. Probability of Detection for the Proposed DM Method

**Figure 10:**
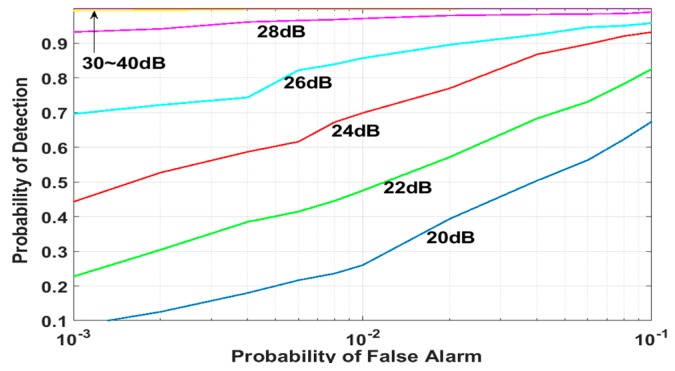
Probability of detection obtained for the proposed DM method (C/N_0_): 33-chip delay.

For the proposed DM acquisition method as in Figure 5, the probability of detection is shown with respect to the probability of false alarm in Figure 10, where each point in the plot is obtained from 10,000 simulation runs. 

**Figure 11:**
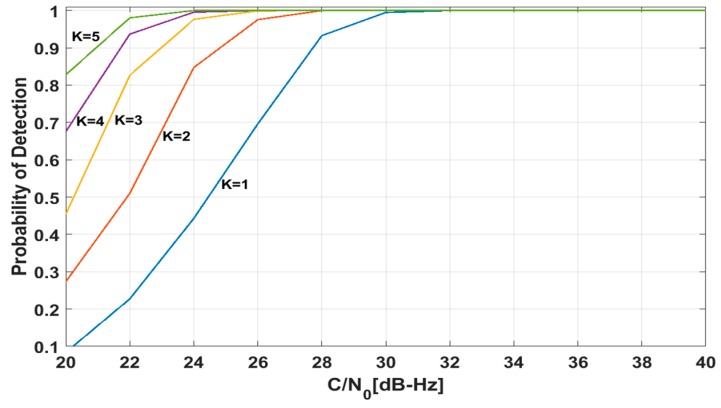
P_d_ with respect to C/N_0_ and *K* for the DM method (P_fa_ = 0.001).

Figure 11 shows the probability of detection according to C/N_0_ with P_fa_ = 0.001. As discussed in Section 3.2.4, the detection probability can be increased by sampling more fast as well as by using longer samples like non-coherent integration. Figure 11 shows the detection probability with using longer samples, i.e., non-coherent integration. If we use $KT_{c}$ long samples, where K is the number of non-coherent integration and $T_{c}$ is the coherent integration time, the detection performance of DM method can be improved. As K increases, P_d_ also increases as in Figure 11.

#### 3.3.3. Finding the Doppler Frequency

In this section we obtain the Doppler frequency after finding the code phase as in Section 3.1.3. In Figure 7, after the code phase aligned, the multiplication of the received signal r(t) and $g_{conv,P}\left(t\right)$ contains the Doppler frequency $f_{D}$ as:

(24) $$r\left(t\right)\cdot g_{conv,P}\left(t\right)\approx \pm \frac{1}{2}\sqrt{\frac{3}{2}c}\cdot \cos\left(2\pi f_{D}t\right).$$

**Figure 12:**
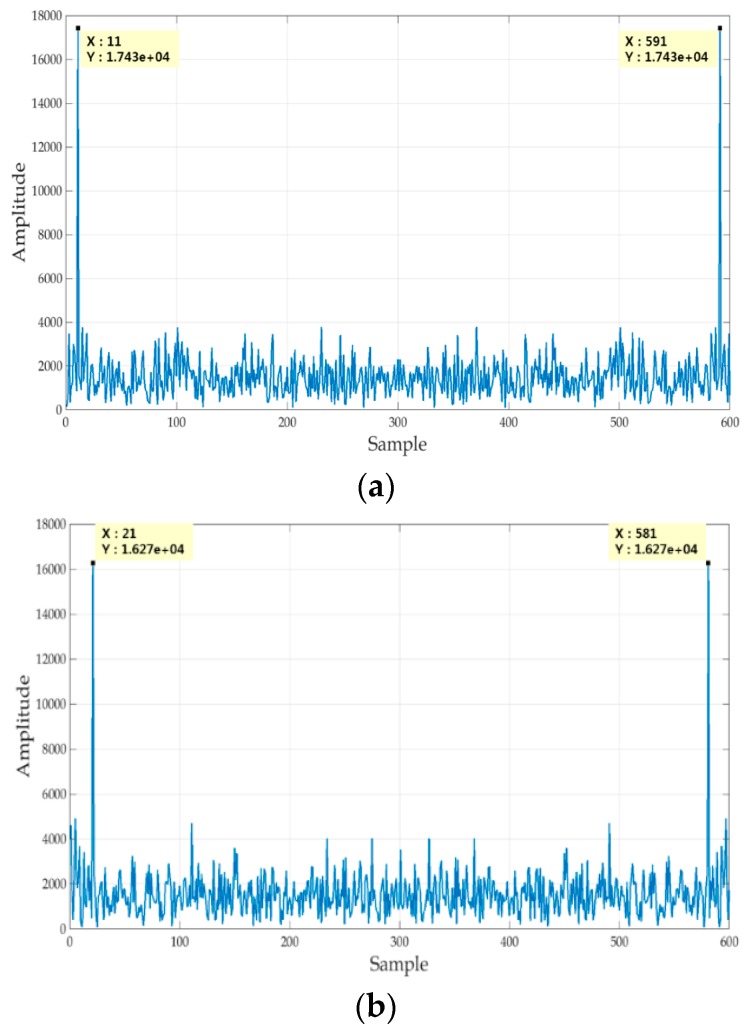

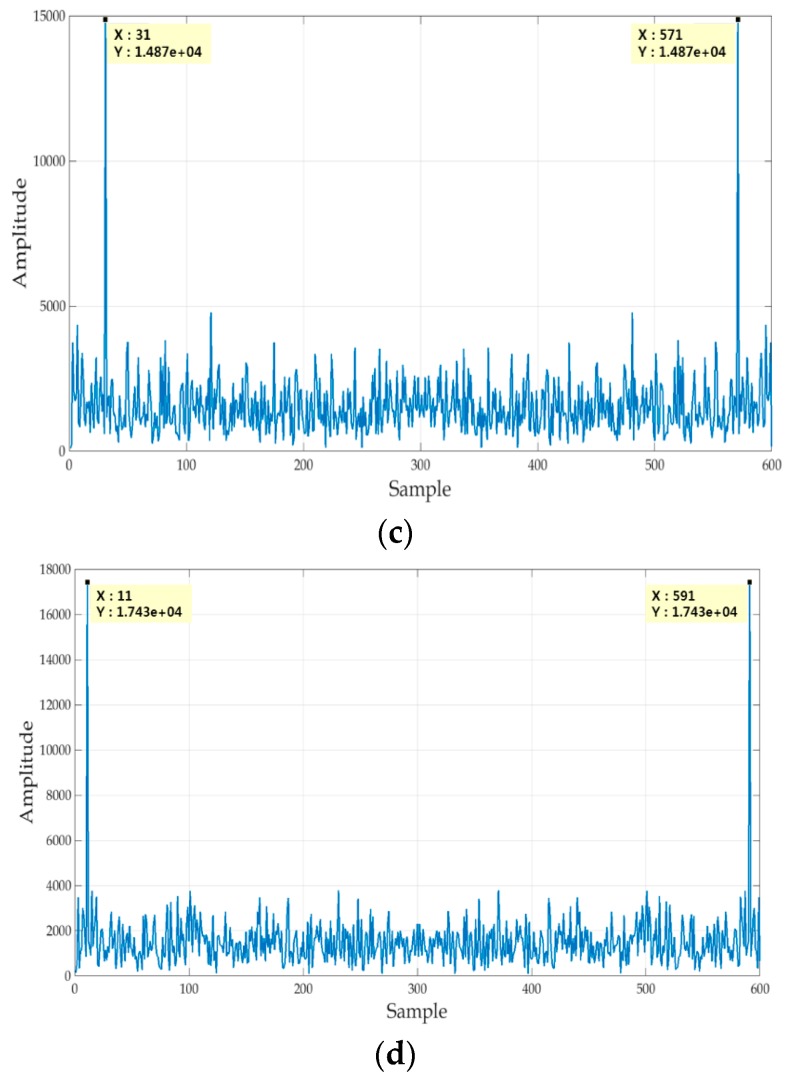
FFT results; (**a**) $f_{D}=1\;\mathrm{kHz};$ (**b**) $f_{D}=2\;\mathrm{kHz};$ (**c**) $f_{D}=3\;\mathrm{kHz};$ (**d**) $f_{D}=-1\;\mathrm{kHz};$

Thus we can obtain $f_{D}$ using the FFT method. We use the received signal r(t) for 10 ms with 600,000 samples, i.e., M= 600,000 and multiply it with $g_{conv,P}\left(t\right)$, and then average 1000 samples resulting in $\bar{M}=$ 600 samples. The frequency resolution of the FFT is 100 Hz since 10 ms data is used. Figure 12 shows the FFT result from the 600 samples with Doppler frequencies, such as 1 kHz, 2 kHz, 3 kHz, and −1 kHz. We can notice that the cases of $f_{D}=1\;\mathrm{kHz}$ and $f_{D}=-1\;\mathrm{kHz}$ show the same result because of cosine function in (24).

### 3.4. Modified DM Acquisition for GPS L1C Signal Using Combined L1/L1C Signal Acquisition

In Section 3.1.1 the optimal delay is chosen as $\sigma_{0}=33T_{0}$ for the DM signal and the magnitude of the received DM signal $s_{DM,\sigma_{0}\;}\left(t\right)$ is given as $d_{0}=\pm \frac{c}{4}\cos\left(2\pi f_{D}\sigma_{0}\right)$ in Equations (10) and (13). This optimal delay $\sigma_{0}=33T_{0}$ is so long that the Doppler frequency effect cannot be ignored in $d_{0}=\pm \frac{c}{4}\cos\left(2\pi f_{D}\sigma_{0}\right)$ as discussed in Section 3.1.1, and thus the proposed DM methods in Section 3.1.2 can be used only for low speed up to approximately 200 km/h.

In this section we propose a modified DM acquisition method which can be used even for high speed and high dynamics. Suppose that we obtain the Doppler frequency in advance by using the L1 signal acquisition.

Define the generated signal $g_{D}\left(t\right)=\cos\left(2\pi \left(f_{IF}+{\hat{f}}_{D}\right)t\right),$ where ${\hat{f}}_{D}$ is the Doppler frequency obtained from the L1 signal acquisition, and $r_{g}\left(t\right)$ as follows:

(25) $$r_{g}\left(t\right)=r\left(t\right)g_{D}\left(t\right)$$

$$=S_{L1C}\left(t\right)\cos\left(2\pi \left(f_{IF}+f_{D}\right)t\right)\cos\left(2\pi \left(f_{IF}+{\hat{f}}_{D}\right)t\right)\approx \frac{1}{2}S_{L1C}\left(t\right)\cos\left(2\pi \left(f_{D}-{\hat{f}}_{D}\right)t\right)$$

where the high frequency term is considered as removed during the correlation process.

The DM signal of $r_{g}\left(t\right)$ with the optimal delay is given as follows:

$$s_{gDM,\sigma_{0}\;}\left(t\right)=r_{g}\left(t\right)r_{g}\left(t-\sigma_{0}\right)\approx S_{L1C}\left(t\right)S_{L1C}\left(t-\sigma_{0}\right)\times \frac{1}{4}\cos\left(2\pi \left(f_{D}-{\hat{f}}_{D}\right)\sigma_{0}\right).$$

If the Doppler frequency error is 100 Hz, then $\cos\left(2\pi \left(f_{D}-{\hat{f}}_{D}\right)\sigma_{0}\right)=0.998,$ and thus the magnitude of the received DM signal $s_{gDM,\sigma_{0}\;}\left(t\right)$ can be considered as constant regardless of the Doppler frequency. We can substitute the $r_{g}\left(t\right)$ in Equation (25) for r(t) Figure 5a and Figure 6a as a modified DM acquisition method.

## 4. Discussion and Conclusions

This paper considers the acquisition problem of the GPS L1C signal. One difficulty of GPS L1C signal acquisition is the ambiguity problem, which comes from the multiple peaks in the correlation function between the received signal and the generated signal. We use the DM method to solve the ambiguity problem and propose two unambiguous DM acquisition schemes for GPS L1C signal. We have chosen the optimal delay $\delta_{0}$ of the DM signal as 33-chip delay of the ranging code with the condition of $\cos\left(2\pi f_{IF}\delta_{0}\right)=\pm 1$. We have proven that by using our proposed DM methods the correlation function between the received signal and the generated signal has a triangular shape with respect to the code phase as in the correlation function of GPS L1 C/A code, which completely removes the ambiguity problem.

However, this 33-chip delay is so long that the Doppler frequency has an effect on the magnitude of the DM signal and thus the proposed DM method can be used for moving receivers up to approximately 200 km/h, for which the magnitude of the DM signal may decrease to the half due to the Doppler frequency for the worst case. One remedy for the magnitude decrease of DM signal is to increase the sampling speed. Faster sampling can recover the SNR and increases the probability of detection as proved in Lemma 7. In the case of the use of L1C signal only the proposed DM method can be used only for low speed application. Second remedy is to use the acquisition result of the legacy GPS L1 signal, which will provide the Doppler frequency. GPS receivers can receive both L1 and L1C signal together and thus the limitation of the DM method can be solved by using the combined L1/L1C acquisition method.

We also analyze the performance of the proposed DM method. The SNR of the DM acquisition method and that of the conventional method are derived analytically and the detection performance of the two methods is compared. The probability of detection is used as the performance measure in the acquisition process, with fixed false alarm probability. The performance result of the proposed DM acquisition methods is shown via simulations.

## Figures and Tables

**Table 1:** Comparison of the performance between the DM method and the conventional method.

	Delay-And-Multiply Method		Conventional Method		
*g(t)*	$g_{DM,PP}$	$g_{DM,PP+DD}$	$g_{conv,P}$	$g_{conv,\sqrt{3}P+D}$	$g_{conv,P+D}$
$Z_{H_{0}}$	$N\left(0,\;M\sigma_{n}^{4}\right)$	$N\left(0,\;2M\sigma_{n}^{4}\right)$	$N\left(0,\;\frac{M}{2}\sigma_{n}^{2}\right)$	$N\left(0,\;2M\sigma_{n}^{2}\right)$	$N\left(0,\;M\sigma_{n}^{2}\right)$
$Z_{H_{1}}$	$N\left(\frac{3c}{4}\cos\left(2\pi f_{D}\delta_{0}\right)M,\;\;\left(\sigma_{n}^{4}+2c\sigma_{n}^{2}\right)M\right)$	$N\left(c\cdot \cos\left(2\pi f_{D}\delta_{0}\right)M,\;\;\left(2\sigma_{n}^{4}+4c\sigma_{n}^{2}\right)M\right)$	$N\left(\pm \sqrt{\frac{3c}{8}}M,\;\frac{1}{2}\sigma_{n}^{2}M\right)$	$N\left(\pm \sqrt{\frac{c}{2}}M,\;2\sigma_{n}^{2}M\right)$ or $N\left(\pm \sqrt{2c}M,\;2\sigma_{n}^{2}M\right)$	$N\left(\pm \sqrt{\frac{c}{2}}M,\;\sigma_{n}^{2}M\right)$ or $N\left(0,\;\sigma_{n}^{2}M\right)$
SNR for $Z_{H_{1}}$	$\frac{\frac{9}{16}\cdot c^{2}\cos^{2}\left(2\pi f_{D}\delta_{0}\right)M}{\sigma_{n}^{4}+2c\sigma_{n}^{2}}$	$\frac{\frac{1}{2}\cdot c^{2}\cos^{2}\left(2\pi f_{D}\delta_{0}\right)M}{\sigma_{n}^{4}+2c\sigma_{n}^{2}}$	$\frac{\frac{3}{4}c\cdot M}{\sigma_{n}^{2}}$	$\frac{\frac{1}{4}c\cdot M}{\sigma_{n}^{2}}$ or $\frac{c\cdot M}{\sigma_{n}^{2}}$	$\frac{\frac{1}{2}c\cdot M}{\sigma_{n}^{2}}$ or 0
preference	o		o		

## Appendix A

### The Proof of Lemma 5

The proof is similar to that of Lemma 4, and thus we use the same notation as in Lemma 4. The difference is that here, the generated signal is $g_{DM,PP+DD}\left(t\right)=C_{PP,\delta_{0}}\left(t\right)+C_{DD,\delta_{0}}\left(t\right)$, instead of $g_{DM,PP}\left(t\right)=C_{PP,\delta_{0}}\left(t\right).$

Consider the $H_{0}$ hypothesis:

$$\begin{array}{ll} H_{0}\;:\;\;\;Z_{H_{0}} & =\overset{M-1}{\underset{i=0}{\sum }}n\left(t_{i}\right)n\left(t_{i}-33T_{0}\right)\{C_{PP,\delta_{0}}\left(t\right)+C_{DD,\delta_{0}}\left(t\right)\}\\ & =\overset{M-1}{\underset{i=0}{\sum }}n_{DM,\delta_{0}}\left(t\right)C_{PP,\delta_{0}}\left(t\right)+\overset{M-1}{\underset{i=0}{\sum }}n_{DM,\delta_{0}}\left(t\right)C_{DD,\delta_{0}}\left(t\right) \end{array}$$

Then we have the Gaussian distribution for $H_{0}\;\mathrm{as}\;Z_{H_{0}}\;\sim \;N\left(0,\;2M\sigma_{n}{}^{4}\right)$.

Suppose that the GPS L1C signal is present, and that the received signal and the generated signal are aligned in phase; the decision variable then becomes:

$$\begin{array}{ll} H_{1}\;\;:\;\;Z_{H_{1}} & =\sum_{i=0}^{M-1}r_{DM,\delta_{0}}\left(t_{i}\right)\;\{C_{PP,\delta_{0}}\left(t_{i}\right)+C_{DD,\delta_{0}}\left(t_{i}\right)\}\\ & =\sum_{i=0}^{M-1}s_{DM,\delta_{0}}\left(t_{i}\right)\;\;\{C_{PP,\delta_{0}}\left(t_{i}\right)+C_{DD,\delta_{0}}\left(t_{i}\right)\}\\ & +\sum_{i=0}^{M-1}S_{L1C}\left(t_{i}\right)\cos\left(2\pi \left(f_{IF}+f_{D}\right)t_{i}\right)\;n\left(t_{i}-\delta_{0}\right)\;\{C_{PP,\delta_{0}}\left(t_{i}\right)+C_{DD,\delta_{0}}\left(t_{i}\right)\}\\ & +\sum_{i=0}^{M-1}S_{L1C}\left(t_{i}-\delta_{0}\right)\cos\left(2\pi \left(f_{IF}+f_{D}\right)(t_{i}-\delta_{0}\right)\;n\left(t_{i}\right)\;\{C_{PP,\delta_{0}}\left(t_{i}\right)+C_{DD,\delta_{0}}\left(t_{i}\right)\}\\ & +\sum_{i=0}^{M-1}n_{DM,\delta_{0}}\left(t_{i}\right)\;\{C_{PP,\delta_{0}}\left(t_{i}\right)+C_{DD,\delta_{0}}\left(t_{i}\right)\}. \end{array}$$

The first term is given using Equation (16) as follows:

$$\begin{array}{ll} Z_{H_{1,1}} & =\sum_{i=0}^{M-1}s_{DM,\delta_{0}}\left(t_{i}\right)\;\;\{C_{PP,\delta_{0}}\left(t_{i}\right)+C_{DD,\delta_{0}}\left(t_{i}\right)\}\approx \;3d_{0}\sum_{i=0}^{M-1}C_{PP,\delta_{0}}\left(t_{i}\right)C_{PP,\delta_{0}}\left(t_{i}\right)+d_{0}\sum_{i=0}^{M-1}C_{DD,\delta_{0}}\left(t_{i}\right)C_{DD,\delta_{0}}\left(t_{i}\right)\\ & =4d_{0}M=c\cdot \cos\left(2\pi f_{D}\delta_{0}\right)M. \end{array}$$

The second term $Z_{H_{1,2}}$ has the Gaussian distribution with mean zero and variance $\sigma_{Z_{H_{1,2}}}^{2}$, which is given as follows using the similar procedure in the proof of Lemma 4:

$$\sigma_{Z_{H_{1,2}}}^{2}=\sum_{i=0}^{M-1}\;\left\{S_{L1C}^{2}\left(t_{i}\right)\cos^{2}\left(2\pi \left(f_{IF}+f_{D}\right)t_{i}\right)\{C_{PP,\delta_{0}}^{2}\left(t_{i}\right)+C_{DD,\delta_{0}}^{2}\left(t_{i}\right)\right\}\}\sigma_{n}^{2}\approx 2cM\sigma_{n}^{2}$$

Similarly, we obtain that $E\left[Z_{H_{1,3}}\right]=0,\sigma_{Z_{H_{1,3}}}^{2}\approx 2cM\sigma_{n}^{2}.$ Also $E\left[Z_{H_{1,4}}\right]=0,\;\sigma_{Z_{H_{1,4}}}^{2}=M\sigma_{n}^{4}.$

In summary, the decision variable has a Gaussian distribution, as follows:

$$Z_{H_{1}}\sim N\left(c\cdot \cos\left(2\pi f_{D}\delta_{0}\right)M,\;\;\left(2\sigma_{n}^{4}+4c\sigma_{n}^{2}\right)M\right).$$

Thus the SNR is obtained as follows:

$$SNR_{DM}=\frac{E{\left[Z_{H_{1}}\right]}^{2}}{\sigma_{H_{1}}^{2}}=\frac{{\left(c\cdot \cos\left(2\pi f_{D}\delta_{0}\right)M\right)}^{2}}{2\left(\sigma_{n}^{4}+2c\sigma_{n}^{2}\right)M}=\frac{\frac{1}{2}\cdot c^{2}\cos^{2}\left(2\pi f_{D}\delta_{0}\right)M}{\sigma_{n}^{4}+2c\sigma_{n}^{2}}$$

## Appendix B

### The Proof of Lemma 6

Consider the received signal r(t) of the GPS L1C signal in Equation (4), and the three cases of generated signal $g_{conv,i}\left(t\right)$. We use the same notation and similar procedure as in the proof of Lemma 4, and the Doppler frequency *f_D_* = 0 from the assumption:

$$\begin{array}{l} \left(i\right)\;g_{conv,P}\left(t\right)=C_{P}\left(t\right)SC_{P}\left(t\right)\cos\left(2\pi f_{IF}t\right).\\ \;\;\;\;H_{0}:\;\;\;Z_{H_{0}}=\sum_{i=0}^{M-1}n\left(t_{i}\right)C_{P}\left(t_{i}\right)SC_{P}\left(t_{i}\right)\cos\left(2\pi f_{IF}t_{i}\right)\;\;\;\;E[Z_{H_{0}}]=0,\\ \;\;\;\;\;\;\;\;\;\;\;\sigma_{Z_{H_{0}}}^{2}=\sum_{i=0}^{M-1}\;\sigma_{n}^{2}{\left(C_{P}\left(t_{i}\right)\right)}^{2}{\left(SC_{P}\left(t_{i}\right)\right)}^{2}\cos^{2}\left(2\pi f_{IF}t_{i}\right)\approx \;\sigma_{n}^{2}\sum_{i=0}^{M-1}1\cdot 1\cdot \frac{1}{2}=\frac{1}{2}\sigma_{n}^{2}M, \end{array}$$

that is, $Z_{H_{0}}\;\sim \;N\left(0,\;\frac{1}{2}\sigma_{n}^{2}M\right)$.

$$H_{1}\;:\;Z_{H_{1}}=\sum_{i=0}^{M-1}S_{L1C}\left(t_{i}\right)\cos\left(2\pi f_{IF}t_{i}\right)\cdot C_{P}\left(t_{i}\right)SC_{P}\left(t_{i}\right)\cos\left(2\pi f_{IF}t_{i}\right)\;+\sum_{i=0}^{M-1}n\left(t_{i}\right)C_{P}\left(t_{i}\right)SC_{P}\left(t_{i}\right)\cos\left(2\pi f_{IF}t_{i}\right)\;\;$$

$$=\sum_{i=0}^{M-1}\begin{array}{c} \left\{\sqrt{\frac{3}{2}c}\cdot C_{OL}\left(t_{i}\right)C_{P}\left(t_{i}\right)SC_{P}\left(t_{i}\right)+\sqrt{\frac{c}{2}}\cdot D\left(t_{i}\right)C_{D}\left(t_{i}\right)SC_{D}\left(t_{i}\right)\right\}C_{P}\left(t_{i}\right)SC_{P}\left(t_{i}\right)cos^{2}\left(2\pi f_{IF}t_{i}\right)+Z_{H_{0}} \end{array}$$

$$\approx \sum_{i=0}^{M-1}\begin{array}{c} \left\{\sqrt{\frac{3}{2}c}\cdot C_{OL}\left(t_{i}\right)C_{P}^{2}\left(t_{i}\right)SC_{P}^{2}\left(t_{i}\right)\cos^{2}\left(2\pi f_{IF}t_{i}\right)\right\}+Z_{H_{0}} \end{array}\;=\sum_{i=0}^{M-1}\begin{array}{c} \left\{\sqrt{\frac{3}{2}c}\cdot \pm 1\cdot 1\cdot 1\cdot \frac{1}{2}\right\}+Z_{H_{0}} \end{array}=\pm \sqrt{\frac{3c}{8}}M+Z_{H_{0}}.$$

that is, $Z_{H_{1}}\;\sim \;N\left(\pm \sqrt{\frac{3c}{8}}M,\;\frac{1}{2}\sigma_{n}^{2}M\right)$.

Thus, the SNR is obtained as follows:

$$SNR_{Conv,P}=\frac{E{\left[Z_{H_{1}}\right]}^{2}}{\sigma_{H_{1}}^{2}}=\frac{\frac{3c}{4}M}{\sigma_{n}^{2}}.$$

(ii) $g_{conv,\sqrt{3}P+D}\left(t\right)=\left(\sqrt{3}C_{P}\left(t\right)SC_{P}\left(t\right)+C_{D}\left(t\right)SC_{D}\left(t\right)\right)\cos\left(2\pi f_{IF}t\right).$

For the $H_{0}$ hypothesis:

$$\begin{array}{l} H_{0}:\;\;\;\;Z_{H_{0}}=\sum_{i=0}^{M-1}n\left(t_{i}\right)\left(\sqrt{3}C_{P}\left(t\right)SC_{P}\left(t\right)+C_{D}\left(t\right)SC_{D}\left(t\right)\right)\cos\left(2\pi f_{IF}t_{i}\right)\;\;\;\;E[Z_{H_{0}}]=0,\\ \;\;\;\;\;\;\;\;\;\;\sigma_{Z_{H_{0}}}^{2}=\sum_{i=0}^{M-1}\;\sigma_{n}^{2}{\left(\sqrt{3}C_{P}\left(t\right)SC_{P}\left(t\right)+C_{D}\left(t\right)SC_{D}\left(t\right)\right)}^{2}\cos^{2}\left(2\pi f_{IF}t_{i}\right)\\ \;\;\;\approx \sigma_{n}^{2}\sum_{i=0}^{M-1}\left\{{\left(\sqrt{3}C_{P}\left(t\right)SC_{P}\left(t\right)\right)}^{2}+{\left(C_{D}\left(t\right)SC_{D}\left(t\right)\right)}^{2}\right\}\cdot \frac{1}{2}=\left(3+1\right)\cdot \frac{1}{2}M\sigma_{n}^{2}=2M\sigma_{n}^{2}, \end{array}$$

we obtain that $Z_{H_{0}}\;\sim \;N\left(0,\;2\sigma_{n}^{2}M\right)$.

$$H_{1}:\;Z_{H_{1}}=\overset{M-1}{\underset{i=0}{\sum }}S_{L1C}\left(t_{i}\right)\cos\left(2\pi \left(f_{IF}\right)t_{i}\right)\left\{\sqrt{3}C_{P}\left(t_{i}\right)SC_{P}(t_{i}\right)+C_{D}\left(t_{i}\right)SC_{D}\left(t_{i}\right)\}\cos\left(2\pi f_{IF}t_{i}\right)+Z_{H_{0}}\;$$

$$\begin{array}{l} E[Z_{H_{1}}]=\sum_{i=0}^{M-1}\left\{\sqrt{\frac{3}{2}c}\cdot C_{OL}\left(t_{i}\right)C_{P}\left(t_{i}\right)SC_{P}\left(t_{i}\right)+\sqrt{\frac{c}{2}}\cdot D\left(t_{i}\right)C_{D}\left(t_{i}\right)SC_{D}\left(t_{i}\right)\right\}\left\{\sqrt{3}C_{P}\left(t_{i}\right)SC_{P}\left(t_{i}\right)+C_{D}\left(t_{i}\right)SC_{D}\left(t_{i}\right)\right\}\cos^{2}\left(2\pi f_{IF}t_{i}\right)\\ \;\;\;\;\;\;\;\;\;\;\;\;\;\approx \sqrt{\frac{c}{2}}\sum_{i=0}^{M-1}\{3C_{OL}\left(t_{i}\right)C_{P}^{2}\left(t_{i}\right)SC_{P}^{2}\left(t_{i}\right)+D\left(t_{i}\right)C_{D}^{2}\left(t_{i}\right)SC_{D}^{2}\left(t_{i}\right)\}\frac{1}{2}=\sqrt{\frac{c}{2}}\left(\pm 3\pm 1\right)\cdot \frac{1}{2}\cdot M\\ \;\;\;\;\;\;\;\;\;\;\;\sigma_{Z_{H_{1}}}^{2}=\sigma_{Z_{H_{0}}}^{2}=2\sigma_{n}^{2}M \end{array}$$

that is, $Z_{H_{1}}\;\sim \;N\left(\pm \sqrt{\frac{c}{2}}M,\;2\sigma_{n}^{2}M\right)$ or $N\left(\pm \sqrt{2c}M,\;2\sigma_{n}^{2}M\right)$

Thus, the SNR is obtained as follows:

$$SNR_{conv,\sqrt{3}P+D}=\frac{E{\left[Z_{H_{1}}\right]}^{2}}{\sigma_{H_{1}}^{2}}=\frac{\frac{1}{4}c\cdot M}{\sigma_{n}^{2}}\;\mathrm{or}\;\frac{c\cdot M}{\sigma_{n}^{2}}$$

(iii) $g_{conv,P+D}\left(t\right)=\left(C_{P}\left(t\right)SC_{P}\left(t\right)+C_{D}\left(t\right)SC_{D}\left(t\right)\right)\cos\left(2\pi f_{IF}t\right).$

By the similar procedure as in (ii), we obtain that:

$$Z_{H_{0}}\;\;\sim \;N\left(0,\;\sigma_{n}^{2}M\right),\;\mathrm{and}\;\;Z_{H_{1}}\;\sim \;N\left(\pm \sqrt{\frac{c}{2}}M,\;\sigma_{n}^{2}M\right)\;\;\mathrm{or}\;\;N\left(0,\;2\sigma_{n}^{2}M\right)$$

Thus, the SNR is obtained as follows:

$$SNR_{conv,P+D}=\frac{E{\left[Z_{H_{1}}\right]}^{2}}{\sigma_{H_{1}}^{2}}=\frac{\frac{1}{2}c\cdot M}{\sigma_{n}^{2}}\;\mathrm{or}\;0$$
